# Heterogeneous Photocatalysis of Metronidazole in Aquatic Samples

**DOI:** 10.3390/molecules26247612

**Published:** 2021-12-15

**Authors:** Klaudia Stando, Patrycja Kasprzyk, Ewa Felis, Sylwia Bajkacz

**Affiliations:** 1 Department of Inorganic Chemistry, Analytical Chemistry and Electrochemistry, Faculty of Chemistry, Silesian University of Technology, B. Krzywoustego 6 Str., 44-100 Gliwice, Poland; kasprzyk.patrycja@interia.pl (P.K.); sylwia.bajkacz@polsl.pl (S.B.); 2 Centre for Biotechnology, Silesian University of Technology, B. Krzywoustego 8 Str., 44-100 Gliwice, Poland; ewa.felis@polsl.pl; 3 Department of Environmental Biotechnology, Faculty of Power and Environmental Engineering, Silesian University of Technology, Akademicka 2 Str., 44-100 Gliwice, Poland

**Keywords:** advanced oxidation process, heterogeneous photocatalysis, metronidazole, degradation pathway, semiconductor catalyst

## Abstract

Metronidazole (MET) is a commonly detected contaminant in the environment. The compound is classified as poorly biodegradable and highly soluble in water. Heterogeneous photocatalysis is the most promoted water purification method due to the possibility of using sunlight and small amounts of a catalyst needed for the process. The aim of this study was to select conditions for photocatalytic removal of metronidazole from aquatic samples. The effect of catalyst type, mass, and irradiance intensity on the efficiency of metronidazole removal was determined. For this purpose, TiO_2_, ZnO, ZrO_2_, WO_3_, PbS, and their mixtures in a mass ratio of 1:1 were used. In this study, the transformation products formed were identified, and the mineralization degree of compound was determined. The efficiency of metronidazole removal depending on the type of catalyst was in the range of 50–95%. The highest MET conversion (95%) combined with a high degree of mineralization (70.3%) was obtained by using a mixture of 12.5 g TiO_2_–P25 + PbS (1:1; *v*/*v*) and running the process for 60 min at an irradiance of 1000 W m^−2^. Four MET degradation products were identified by untargeted analysis, formed by the rearrangement of the metronidazole and the C-C bond breaking.

## 1. Introduction

The possibility of reusing gray water and wastewater is the main goal of rational water management. For this purpose, it is necessary to develop new effective water and wastewater treatment technologies. Conventionally used wastewater treatment consists of three stages: primary treatment (filtration, floatation, sedimentation), secondary treatment (biological and/or chemical treatment), and complementary treatment (filtration, coagulation, adsorption processes, flocculation, or purification by means of more advanced techniques) [1]. Conventional purification methods are insufficient to remove xenobiotics, i.e., antibiotics, pesticides, and household chemicals [2,3]. An alternative to conventional purification methods or their supplement after the conventional process are the advanced oxidation processes (AOPs). In AOPs, non-selective degradation and mineralization of organic pollutants occur due to the action of hydroxyl radicals (-OH) [4]. AOPs can include ozonation, Fenton and photo-Fenton, photolysis, heterogeneous photocatalysis with semiconductors, and electrochemical processes [5]. Depending on the type of process, hydroxyl radicals can be formed from compounds such as ozone (ozonation), hydrogen peroxide (Fenton processes), oxygen, and water (photocatalytic processes) [6,7]. The main advantages attributed to AOPs are their fast reaction rate, non-selectivity in pollutant removal, and their ability to be combined with biological treatment as an additional water treatment step before discharge to the environment. However, there are several drawbacks which means that not all of the mentioned AOPs processes can be used in industrial settings, such as high consumption of energy and large amounts of chemical reagents needed [8]. In particular, Fenton processes, which require acidification of the environment and the presence of H_2_O_2_, can be harmful to humans and other living organisms [9,10].

The most promising AOPs technique in terms of environmental and economic aspects is heterogeneous photocatalysis using semiconductor catalysts. In heterogeneous photocatalysis, the only requirement is the presence of a catalyst that will generate hydroxyl radicals due to light absorption [11]. Additionally, compared with homogeneous catalysis, heterogeneous catalysis is superior owing to several key advantages: stability, easy separation, and reusability of the catalyst [12]. In general terms, heterogeneous photocatalysis involves the excitation of a valence electron ($e^{-}$) into the conduction band, followed by a surface catalysis step where photoinduced electron-hole pairs are involved in the reduction of compounds adsorbed on the surface [13]. The key step required to initiate the redox reaction is the transfer of reactants present in the liquid phase to the catalyst surface and their adsorption [14]. Valence band holes are strong oxidants and react with water or hydroxyl ions to form hydroxyl radicals, while conduction band electrons are reducers and react with dissolved oxygen to form superoxide anion radical (O_2_*^•−^*), which then converts to hydrogen peroxide, which decomposes into hydroxyl radicals [5,15].

N-type semiconductors with narrow band gap and suitable band edge capabilities are used as photocatalysts. Titanium dioxide (TiO_2_) is the most commonly used photocatalyst due to its good photocatalytic performance, stability, and narrow band gap in the range of 3.0–3.2 eV [16]. TiO_2_ in the anatase form exhibits the highest photocatalytic activity due to its extended electron-hole recombination time, high porosity, and surface area [17]. A commercially used form of TiO_2_ is Degussa P25 nanopowder consisting of 75% anatase and 25% rutile, with a surface area of 45 m^2^ g^−1^ [18,19]. For the photodegradation of pharmaceutical residues, in addition to TiO_2_, other semiconductors such as tungsten(VI) oxide [20,21], ZnO [22,23], Cu_2_O [24,25], and BiWO_6_ [26,27] and BiVO_4_ [28,29] have also been verified.

In the photodegradation process, it is necessary to monitor both the analyte loss over time and to determine the degree of the mineralization [30,31]. For stable extended organic structures, they can degrade with the formation of degradation products (DPs) [16]. If the photocatalysis process lasts long enough, it is possible to fully mineralize the organic residues in the sample; however, this would be energy and time consuming [32,33]. Degradation products in photocatalysis reactions are formed by hydroxyl radical attack on the structure and breaking of C-C, C-N, C-S bonds, and ring rearrangement or hydroxylation [16,34,35].

The aim of this study was to evaluate the removal efficiency of metronidazole (MET) by heterogeneous photocatalysis using different types of semiconductors (TiO_2_, ZnO, WO_3_, ZrO_2_, PbS) and their mixtures. MET is a bactericide and antiprotozoal drug commonly detected in surface waters (rivers, lakes) [36,37] and effluents from wastewater treatment plants [38]. MET is highly stable and non-biodegradable, making it difficult to remove in traditional microbiological treatment [39].

## 2. Materials and Methods

### 2.1. Materials and Methods

Analytical standard of metronidazole (MET) was purchased from Sigma-Aldrich (St. Louis, MO, USA). Two types of titanium(IV) oxide were used in this study—namely Degussa P25 (from Evonik, Essen, Germany) and TiO_2_ in the form of anatase from Sigma-Aldrich (St. Louis, MO, USA). Based on information from the manufacturer, the size of the anatase particles was <25 nm, while the specific surface area ranged from 45 to 55 m^2^ g^−1^. In the case of P25, its particle size was about 27 nm, and the specific surface area was at the level 57 m^2^ g^−1^. The other semiconductor catalysts such as zinc oxide, tungsten(VI) oxide, zircon (IV) oxide, and lead(II) sulphide were purchased from Sigma-Aldrich (St. Louis, MO, USA). Analytical-grade methanol was purchased from CHEMPUR (Piekary Śląskie, Poland). Hypergrade acetonitrile, formic acid, trifluoroacetic acid, water, and methanol were obtained from Merck (Darmstadt, Germany) and used for the LC analyses.

### 2.2. Preparation of the Standard Solutions

The standard stock solution of metronidazole with a concentration of 10 mg mL^−1^ was prepared by weighing on an analytical balance 100 mg of metronidazole standard and dissolving in 10 mL of methanol. Working solutions of MET in the range of 0.05–5.00 µg mL^−1^ were prepared by diluting standard stock solution. An amount of 1.0 µg mL^−1^ MET solution prepared in distilled water was used as a model sample for photocatalytic experiments.

### 2.3. Performance of the Photodegradation Experiments

Six types of semiconductors (TiO_2_, TiO_2_–P25, ZnO, ZrO_2_, WO_3_, PbS) and their nine mixtures (ZnO + ZrO_2_; ZnO + PbS; ZnO + WO_3_; TiO_2_ + ZrO_2_; TiO_2_ + PbS; TiO_2_ + WO_3_; TiO_2_–P25 + ZrO_2_; TiO_2_–P25 + PbS; TiO_2_–P25 + WO_3_) in a weight ratio of 1:1 were checked for the study of photocatalytic efficiency of MET degradation. The weight ratio of the selected catalyst mixtures was selected on the basis of the results of the preliminary tests carried out.

The photodegradation experiments were carried out under laboratory conditions in a photoreactor Solarbox 1500e (Co.fo.me.gra, Milan, Italy) equipped with an air-cooled xenon lamp. The xenon lamp used had a wide range of irradiance regulation in the range from 200 W m^−2^ to 1000 W m^−2^. The details on the photoreactor, lamp characteristics, etc. have been discussed in our previous publication [40], and the lamp spectra in order to various lamp irradiance used in the experiment is presented in Supplementary Materials (Figure S1). As part of the experiment, parameters such as the type (mentioned above) and weight of the catalyst (12.5–50.0 mg; concentration 50–200 mg L^−1^), the intensity of irradiance (250, 500, 750, and 1000 W m^−2^), and the duration of the photocatalytic process (60–90 min) were checked. Inside the photoreactor a magnetic stirrer MS-H280-Pro (Chemland, Szczecin Stargard, Poland) was installed.

After selecting the optimal catalyst mass and lamp irradiance, the procedure was as follows: 50 mg of the selected photocatalyst or their mixture was added to 250 mL of a solution containing MET at a concentration of 1.0 μg mL^−1^. The solution was thoroughly mixed and then a photocatalytic process was carried out for 60–90 min, while the sample was constantly stirred. Sampling was carried out at specified time intervals (0, 5, 15, 30, 45, 60, 90 min), collecting 1.0 mL of solution. The samples were filtered through polyethersulfone filters (0.45 µm) and analyzed by LC-UV and LC-MS/MS. For each type of the catalyst, the experiment was carried out three times.

### 2.4. Total Organic Carbon Determination

Total organic carbon (TOC) was determined in the form of non-purgeable organic carbon (NPOC). An organic carbon analyzer TOC-L/TNM-L with TOC-Control L software (Shimadzu, Japan) was used for the *TOC* determination. The liquid sample before (blank samples) and after the photocatalytic process was acidified to pH < 3.0 with 1.0 M HCl and blown with air at a flow of 200 mL min^−1^. Next, the sample was burned with a platinum catalyst at 720 °C with a continuous carrier gas flow. Air was used as the carrier gas at a flow rate of 150 mL min^−1^ and a pressure of 285 ± 5.0 kPa. Carbon dioxide produced by catalytic combustion of the sample was directed to a non-dispersive infrared sensor (NDIR). The analysis was performed at the temperature of 65 ± 1 °C, and the concentration of CO_2_ obtained as a result of combustion of the sample was proportional to the *TOC* content in the sample. Each of the samples was analyzed in triplicate. The degree of sample mineralization was determined from the formula:

$$MR=\frac{TOC_{i}-TOC}{TOC_{i}}\cdot 100\%$$

where *MR* is Mineralization rate (%); *TOC_i_* is the *TOC* value before photocatalytic process, initial concentration of *TOC* in the sample (mg L^−1^); *TOC* is *TOC* value after the photocatalytic process (mg L^−1^).

### 2.5. Instrumentation and Analytical Conditions

The ultrahigh performance liquid chromatograph (LaChrom Ultra Hitachi, Merck, Hitachi, Tokyo, Japan) comprising an autosampler L-2200, two pumps L-2160, thermostat L-2350U, and UV detector L-2400U was used for the analyses. EZ Chrom Elite software was used to control the chromatographic system. The chromatographic separation of metronidazole was carried out on a ZORBAX SB-C3 column (150 mm × 3.0 mm i.d., 5 μm particle size, Agilent Technologies, Agilent, Santa Clara, CA, USA) at 30 °C. Elution was performed in an isocratic system, where the solvents were 0.05% trifluoroacetic acid in water (A) and acetonitrile (B). The composition of the mobile phase was 85:15 (A:B; *v*/*v*), and flow rate was 1.0 mL min^−1^. The duration of the analysis was 3 min, and the injection volume was 20 µL. The MET determination was carried out at a wavelength of λ = 317 nm.

The Dionex HPLC system (Dionex Corporation, Sunnyvale, CA, USA) coupled with an AB Sciex Q-Trap^®^ 4000 mass spectrometer was also used to determine metronidazole and its degradation products. The HPLC consisted of UltiMate 3000 thermostatted column compartment, UltiMate 3000 autosampler, UltiMate 3000 RS pump, and Chromeleon TM 6.8 software. The same ZORBAX-SB-C3 column was used for the chromatographic separation, which was operated at a temperature of 30 °C, but the composition of the mobile phase was changed. A gradient elution was used where the mobile phase consisted of two solvents: acetonitrile (A) and 0.1% formic acid in water (B). The system was initially 10% Solvent A, which was increased linearly over 5 min to 20%. The flow rate was 1 mL min^−1^, and the injection volume was 2 µL. The QTRAP mass spectrometer with electrospray ionization and Analyst 1.4 software was used. The MET determination was performed using the multiple reaction monitoring mode (MRM) in the positive ion mode. The MRM transitions were optimized on MET standard as follows: *m*/*z* 172.1→128.1 m/z (first transition) and 127.1→82.1 *m*/*z* (second transition). The operating parameters of the mass spectrometer, such as declustering potential (DP), collision energy (CE), collision cell exit potential (CXP), and entrance potential (EP) were also optimized. DP and EP for both MET transitions were 76 and 7, respectively. CE = 21 and CXP = 8 were used for the first MRM transition, while for the second transition they were CE = 33, CXP = 6. The ion source parameters were as follows: temperature (TEM) = 500 °C, ion spray voltage (IS) = 4000 V, collision gas (CAD) = medium, curtain gas (CUR) = 10 psi, ion source gas 1 (GS1) = 60 psi, and ion source gas 2 (GS2) = 50 psi.

### 2.6. Method Validation

For the validation of the developed analytical method, parameters such as linearity, limit of detection (*LOD*), limit of quantification (*LOQ*), accuracy, precision, and linearity were determined. A MET calibration curve was prepared for the determination of linearity. A calibration curve was prepared with six points in the concentration range of 0.05–5.0 µg mL^−1^, followed by measuring the area of the peaks. Using linear regression, the equation of the standard curve was determined, and the linearity was determined on the basis of the correlation coefficient R^2^. *LOQ* was determined as the lowest point on the standard curve and *LOD* was calculated from the Equation (1):

(1) $$LOD\;=\;\frac{LOQ}{3}$$

Accuracy and precision were determined at three concentration levels: high quality control (HQC = 4.0 µg mL^−1^), medium quality control (MQC = 2.0 µg mL^−1^), and low quality control (LQC = 0.1 µg mL^−1^). The precision was determined on the basis of the relative standard deviation (*RSD*) according to the Formula (2):

(2) $$RSD\;=\;\frac{SD}{x_{av.}}\;*\;100\%$$

where *SD* is standard deviation; *x_av_* is the average concentration of the analyte in the sample. Relative error (*RE*), calculated according to the Formula (3), was adopted as a measure of accuracy (3):

(3) $$RE\;=\;\left(\frac{x_{measured}\;-\;x_{expected)}}{x_{expected}}\right)\;\times 100\%$$

where *x_measured_* is determined concentration of the analyte in the sample; *x_expected_* is mean concentration of the analyte.

### 2.7. Determination of Metronidazole Degradation Products

Determination of MET degradation products (DPs) was performed for post-reaction mixtures (after the photodegradation process). The DPs were searched for by two approaches: semi-targeted analysis using pseudo MRM mode (p-MRM) and non-targeted analysis using different MS/MS modes. Information on MET degradation products was selected from the literature, and then literature transitions for DPs were introduced to the method as MRM transitions [41,42,43,44]. The ion source parameters were the same as for targeted analysis. Based on this step, DPs that might be in the sample were selected. In the second step, using a QTRAP system that combined a quadrupole analyzer and a linear ion trap, the full scan spectra were recorded, on the basis of which: (I) the structures of the DPs identified in the first step were confirmed, (II) new DPs were searched for by using retrospective analysis. For the non-targeted analysis, two linear ion trap modes were combined: EMS (Enhanced MS) and EPI (Enhanced Ion Scanning). The combination of modes was possible by using intelligent data acquisition (IDA), where the workflow was as follows: (I) EMS survey scan rapidly screened for all compounds in the sample (II) if the IDA criteria were met, i.e., the compound was within the specified m/z range (50–400 m/z) and the signal had a higher intensity than 1000 cps, an ion trap was triggered (III) and EPI scans rapidly collected high-quality MS/MS data.

## 3. Results and Discussion

### 3.1. Developing Chromatographic Conditions

The first stage of the work was to develop a method for the determination of MET in the liquid samples using the LC-UV method. The selection of chromatographic conditions took into account such MET parameters as lipophilicity (logP = (−)0.30−0.02), partition constant pK_a_ = 2.4, and solubility in organic solvents and water (10 mg mL^−1^, 20 °C) [45]. The stationary phase was a Zorbax SB-C3 chromatographic column, whose bed was a porous silica layer with triisopropylsilane, with high chemical stability over a wide pH and temperature range. This column was also used in previous studies for the separation of pharmaceuticals in this MET [46]. The best chromatographic parameters were obtained using an isocratic elution: acetonitrile:0.05% TFA in water (15:85; *v*/*v*). The use of an isocratic system reduced the analysis time to 3 min, with a retention time (RT) of metronidazole of 1.45 min. The detection was performed using a UV spectrophotometric detector at an analytical light wavelength of λ = 317 nm, where the MET absorption maximum occurred. Figure S2 shows the chromatogram of the standard substance.

### 3.2. Validation of the Analytical Method

The newly developed LC-UV method was characterized by good linearity in the range of 0.05–5.0 µg mL^−1^. The R^2^ coefficient for the MET standard curve was 0.9996. The lowest analyte concentration on the standard curve (i.e., 0.05 µg mL^−1^) was taken as the limit of quantification (LOQ). For calculation of LOD, the relationship that LOQ equals the 3-fold LOD and is 0.017 µg mL^−1^ was used. Precision and accuracy were determined at three concentration levels: high (HQC = 4 µg mL^−1^), medium (MQC = 2 µg mL^−1^), and low (LQC = 0.1 µg mL^−1^). Precision, as determined by relative standard deviation (RSD), was in the range of 1.03–3.60%, where RSD decreased with increasing MET concentration in the sample. The RSD value was <15% by which it could be concluded that the method was accurate. RE was also determined at three concentration levels and was for LQC 6.22%, MQC −1.96%, and HQC −0.85%. For all the control levels tested, RE was <7%; thus, the developed method was accurate.

### 3.3. Selection of Heterogenic Photocatalysis Conditions

The influence of individual parameters on the efficiency of photocatalytic MET degradation was analyzed by studying changes in MET concentration during the process and by determining the kinetic parameters of the reaction. The photocatalysis was carried out according to the procedure described in Section 2.3.

#### 3.3.1. Selection of the Photocatalyst

**Figure 1:**
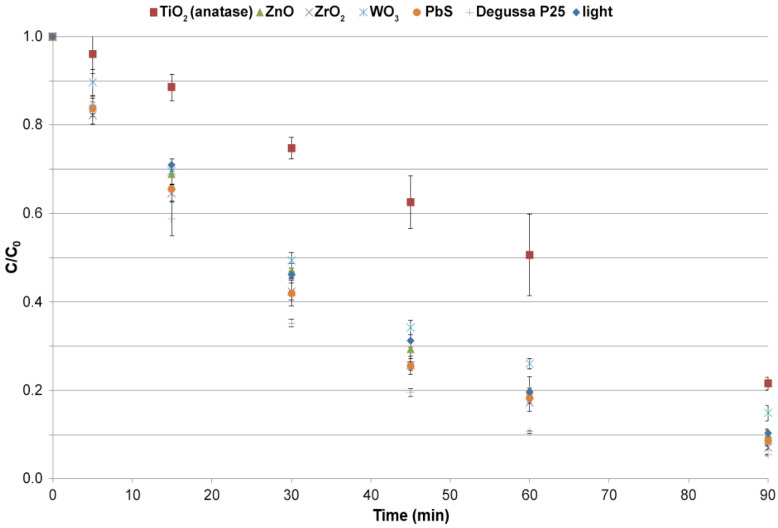
MET removal efficiency during photocatalysis using selected catalysts (catalyst concentration, 50 mg L^−1^; irradiance, 500 W m^−2^; irradiation time, 90 min).

Depending on the type of catalyst, different degrees of its dispersion in water were observed: TiO_2_ (anatase and P-25) and ZnO formed bright milky suspensions throughout the solution. WO_3_ and PbS did not form suspensions and sank to the bottom in the solution, while ZrO_2_ floated on the surface of the solution. It has been shown that the type of dispersion a catalyst forms has a significant effect on its photocatalytic properties [47]. A constant degree of catalyst dispersion throughout the experiment was obtained by continuously stirring the solution during the process. Figure 1 shows a plot of the changes in MET concentration in solution during photocatalysis.

The highest MET removal efficiency (more than 90% MET in 60 min) was shown by TiO_2_ in the form of Degussa P25 nanopowder, while the lowest removal efficiency was observed in the process where TiO_2_ in the form of anatase was used (50% MET removal in 60 min). The low efficiency of TiO_2_ in the anatase form as a catalyst can be justified by its behavior in the dispersing medium. Turbidity affects the optical properties of water and can impede the transmission of UV light [47]. It is observed especially often for nano TiO_2_ in the form of anatase and causes a shielding effect, which alters the light transmission and hinders the access of UV/solar rays [48]. This means that the applied radiation could not fully penetrate the structure of the catalyst molecule and initiate the photooxidation reaction. Thus, the reaction efficiency in this case was influenced not so much by the crystalline form of the catalyst (which in the form of anatase is known to be very photochemically active) but by the problem of activating the catalyst with light, which was limited due to the high turbidity of the reaction solution.

The excellent photocatalytic properties of Degussa P25 are related to the synergistic effect of anatase (75%) and rutile (25%). An additional advantage of this mixture is the fact that both forms of TiO_2_ in the formulation are selected so as not to disturb the parameters related to the turbidity of the reaction solution. In the mixture of two polymorphic varieties of TiO_2_, there is an electron transfer phenomenon from rutile molecules to anatase, which stabilizes the charge separation and slows down electron-hole recombination [49]. The high photocatalytic activity of Degussa P25 is due to its nanostructured properties— more specifically, to its increased catalytic surface area. However, in the case of Degussa P25, constant stirring was necessary due to the tendency of the catalyst particles to form agglomerates [50]. PbS, ZrO_2_, and ZnO showed good photocatalytic properties for MET removal in the range of 80–90% conversion during 90 min. Attempts to use ZnO and ZrO_2_ to remove pharmaceutical contaminants by photocatalytic processes have been reported in the literature. According to the literature, ZrO_2_ is not a good photocatalyst, removing only 4% of amoxicillin in 240 min at an irradiance of 50 W m^−2^ and an initial concentration of 10 mg L^−1^ [21]. Most probably, the discrepancy between the obtained results and the literature data on the catalytic efficiency of ZrO_2_ is due to the different structure of the degraded compound, the lower MET concentration used in the experiment (1 mg L^−1^), and the higher irradiance of 500 W m^−2^. In the case of ZnO, it was shown that under optimized conditions (pH = 11), the efficiency in removing tetracycline is higher than for Degussa P25 [51]. In our study, the pH of the sample was 7, for which Degussa P25 has higher efficiency. The use of lead sulfide as a single catalyst has not been described in the literature so far. It has only been used as a component of composite photocatalysts in AOP [52,53].

In the next part of the research, nine catalyst mixtures (ZnO + ZrO_2_; ZnO + PbS; ZnO + WO_3_; TiO_2_ + ZrO_2_; TiO_2_ + PbS; TiO_2_ + WO_3_; TiO_2_−P25 + ZrO_2_; TiO_2_−P25 + PbS; TiO_2_−P25 + WO_3_) were tested. The selected catalyst mixtures never before have been used to remove pharmaceuticals from liquid samples. There are only single reports that indicate that use of catalysts in the form of binary mixtures can extend the light absorption range if the two components have excited bands with different widths, which will affect the efficiency of photocatalysis [54].

**Figure 2:**
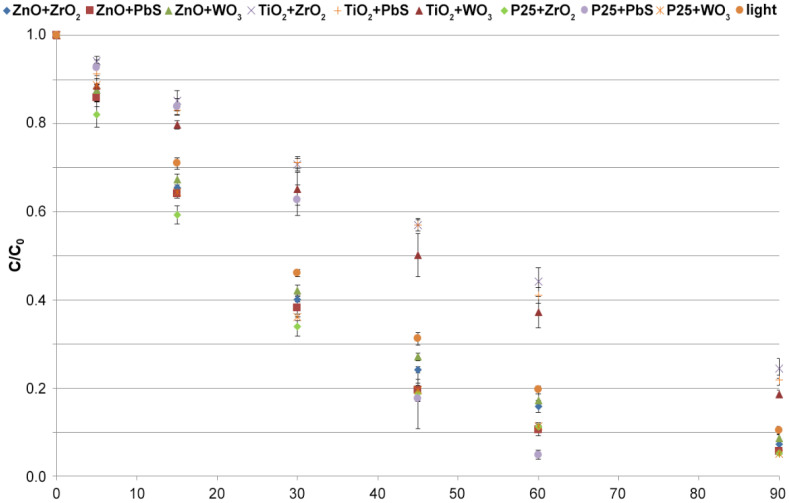
MET removal efficiency during photocatalysis using mixtures of selected catalysts (catalyst concentration, 50 mg L^−1^; irradiance, 500 W m^−2^; irradiation time, 90 min).

The obtained results of MET removal efficiency using the above mixtures are shown in Figure 2. The combination of TiO_2_ with WO_3_, ZrO_2_, and PbS did not significantly enhance the efficiency of the photocatalytic process. A 2.9% increase in MET removal efficiency was observed when TiO_2_ + WO_3_ was used, while a 2.9% decrease was observed for TiO_2_ + ZrO_2_ compared with pure TiO_2_ (Figure S3). For the mixture of ZnO with ZrO_2_ and WO_3_, no significant improvement in MET removal efficiency was observed either, with 2.6% and 1.2% higher at 90 min compared with pure ZnO, respectively. Only for the mixture of ZnO + PbS was it observed that the MET removal efficiency was 10% higher after 60 min of reaction than that of pure ZnO. After 90 min of reaction, the MET conversion was at similar levels for ZnO and ZnO + PbS at 90.1% and 94.4%, respectively (Table S1). The increase in photocatalytic performance with the combination of ZnO + PbS is related to the effect of doping of ZnO by PbS, which results in a change in the electron gap multiplicity [55]. The combination of Degussa P25 with WO_3_ and ZrO_2_ did not change the MET conversion rate. The mixture of Degussa P25 with PbS proved to be the most effective catalyst combination. Using Degussa P25 + PbS (1:1; *w*/*w*), 95% of MET was removed in 60 min, which was only achieved in 90 min with only Degussa P25 (Figure S4).

Table 1 ranks the catalysts and their mixtures with increasing MET degradation rate and the obtained R^2^ determination coefficients. The change in MET concentration over time was assumed to correspond to pseudo-first-order reaction kinetics, and linear regression was used to determine the mathematical model. The R^2^ coefficients were greater than 0.930, confirming a good model fit. TiO_2_ in the form of anatase and its mixtures do not show catalytic properties in the MET photodegradation reaction. The obtained reaction constants are lower (<0.0245) than for the photodegradation reaction using light alone. The process inhibition phenomenon is related to the shielding effect, reducing the active surface area of TiO_2_ and other components exposed to light [56]. The best photocatalyst proved to be the Degussa P25 + PbS combination, where the process proceeded with two reaction rate constants k_1_ up to 20 min and k_2_ 30–60 min, with the second rate constant being much higher than for the other catalysts. Using the Degussa P25 + PbS mixture, MET conversion of 95% can be achieved in 60 min of the process. According to the literature, PbS has a coupling effect with TiO_2_ [57]. Improvement in TiO_2_ photocatalytic properties with sulfur compounds changes the energy gap range and affects the electron flow kinetics and increases the recombination time [55,58]. Considering the values of the rate constants and degradation efficiencies of all photocatalysts, the Degussa P25 + PbS mixture (1:1; *w*/*w*) was selected for further experiments.

#### 3.3.2. Influence of the Catalyst Amount on the Process of Catalysis

**Figure 3:**
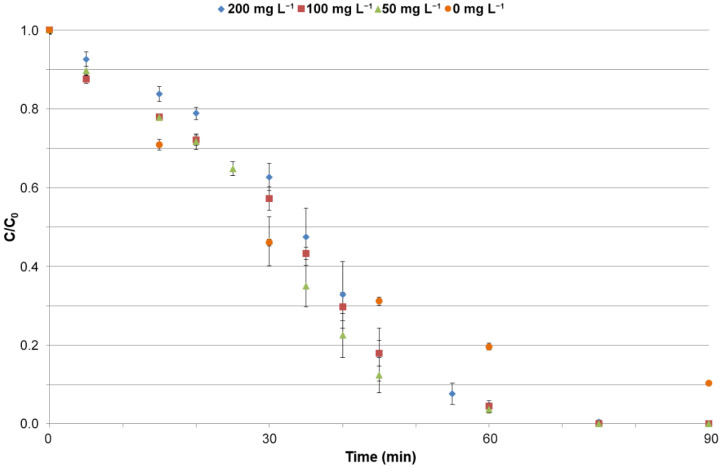
Effect of photocatalyst concentration on metronidazole conversion by photocatalytic process (catalyst, Degussa P25 + PbS (1:1; *w*/*w*); irradiance, 500 W m^−2^; irradiation time, 90 min).

Determining the optimal catalyst mass for the photodegradation process is important for maximizing the degradation rate. At low catalyst mass, there are fewer active sites interacting with the reaction mixture, and hydroxyl radicals form more slowly [59]. Too high catalyst mass causes turbidity of the solution, which leads to light scattering and hinders photon absorption and leads to agglomeration of catalyst particles [60,61]. Figure 3 shows the effect of catalyst weight on MET removal efficiency. After 60 min of using Degussa P25 + PbS catalyst (1:1; *w*/*w*) at concentrations of 50, 100, and 200 mg L^−1^, 96.2%, 95.5%, and 95.3% of METs were removed, respectively. It can be seen that a 4-fold reduction in catalyst weight from the initial concentration of 200 mg L^−1^ does not significantly affect the efficiency of the photocatalytic process. Compared with the control sample, where no catalyst was used only light alone, the Degussa P25 + PbS semiconductor mixture shows catalytic activity. To achieve a MET degradation rate of 95%, it is necessary to irradiate the sample without catalyst for 120 min, the same effect after using a catalyst is achieved after 60 min. Considering the results obtained, a catalyst concentration of 50 mg L^−1^ was used for further studies.

#### 3.3.3. Effect of Irradiance Intensity on the Efficiency of Photocatalysis Process

First, the effect of MET adsorption processes on the catalyst surface was determined, and a dark test (without light irradiation) was performed. Adsorption of the analyte near the active site is one of the steps in heterogeneous photocatalysis, but it can also lead to catalyst deactivation [62]. For the dark test (MET solution with catalyst without light irradiation), no significant loss of MET (1–2%) was observed, indicating that the loss of analyte was not related to its permanent adsorption on the catalyst surface.

The selection of the irradiance range in the experiment was made on the basis of annual solar irradiance data in Poland (temperate, warm transitional climate), where 700 W m^−2^ is the average overcast sky, and 1000 W m^−2^ is a sunny day in summer [63]. According to the SOLARGIS database, the global horizontal irradiance (GHI) for Poland from 1994 to 2018 was in the range of 2.8–3.2 kWh m^−2^, which with an average day length of 12 h gives an irradiance in the range of 233–266 W m^−2^ [64]. Table 2 shows the effect of irradiance on MET removal efficiency.

From the data presented, it can be observed that the time required to remove MET from solution decreases with increasing irradiance. The increase in MET photodegradation efficiency can be explained by the conversion of total irradiance to photon flux, which was involved in the photocatalytic radical generation processes $OH^{∙}$ and $O_{2}^{∙-}$ [65]. The application of irradiance of 250 W m^−2^, corresponding to the daily GHI for Poland, does not allow the complete removal of MET from the solution within 60 min. The highest efficiency was achieved using an irradiance of 1000 W m^−2^, where 95.5% of MET was removed within 30 min. Lowering the irradiance to 500 W m^−2^ resulted in a doubling of the process, as MET conversion of 96.1% was not achieved until the 60th minute. It would be most efficient to use an irradiance of 1000 W m^−2^, but such a value, as already mentioned, is only achievable under environmental conditions during the summer. For efficient MET degradation, it will be necessary to assist with an artificial light source or run the process longer.

#### 3.3.4. *TOC* Studies for Post-Reaction Mixtures

A total organic carbon (TOC) study was conducted to determine the degree of MET mineralization in the post-reaction mixtures. The higher the degree of mineralization, the more efficient the photodegradation process is, because organic pollutants including MET decompose to simple inorganic compounds. Table 3 shows the *TOC* values and the mineralization degree (MD) for the different reaction mixtures.

In order to evaluate the performance of the catalysts, the obtained results were also compared to three kinds of blank samples: *TOCi* (sample before photocatalysis process), MET + UVA (photolysis), and for the best catalyst Degussa P25 + PbS (dark process, without light). For the dark process, the *TOC* value is close to *TOCi*, which confirms that the adsorption process on the catalyst surface does not affect the change in MET content in the liquid phase. Definitely, the factor affecting the MET removal is light, as the MD degree was 52.3% in the 180 min process. The use of single solid-state catalysts allowed mineralization of the sample at a similar degree of 51.5% (TiO_2_-anatase) −62.0% (TiO_2_-P25) in half the time compared with the process without catalyst. The highest degree of mineralization (70.3%) was achieved after 90 min of photocatalysis using the Degussa P25 + PbS mixture, where a 10% increase in MD was observed compared with the pure Degussa P25 (62.0%) and PbS (62.2%) preparation. The MD result obtained in this study is higher compared with the data in the literature on MET photodegradation using other types of photocatalysts: BiOCl/g-C_2_N_4_ (68%) [66], Ag-ZnO/GP (42.1%) [44], D-g-C_3_N_4_-Bi_5_I_7_ (53%) [67]. None of the systems used achieved MD close to 100%, indicating that there are still organic compounds in solution that were not completely removed.

### 3.4. Identification of Degradation Products in Post-Reaction Mixtures

In the photocatalysis process, incomplete degradation of contaminants and formation of degradation products (DPs) may occur [43]. Based on the obtained experimental results, where the removal rate of MET was in the range of 60–95%, while MD was in the range of 36.0–70.3%, it can be assumed that additional DPs are formed during the photodegradation process of MET. A retrospective analysis of the individual mass spectra was then performed to confirm the structure and, where possible, compared with the data in the literature. Table S2 summarizes the identified DPs in the samples after the photocatalysis process using single catalysts and their mixtures.

**Figure 4:**
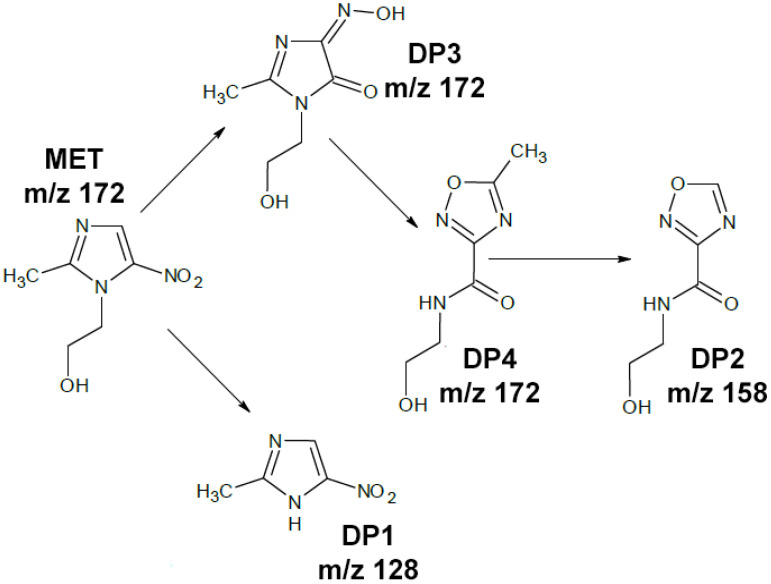
Proposed path of metronidazole photodegradation.

A total of four DPs and MET residues were identified in aquatic samples after photocatalysis. In samples after photodegradation of MET without the use of a catalyst and the use of mixtures of ZnO + WO_3_, ZnO + ZrO_2_, and TiO_2_ + WO_3_, three of these four DPs were detected. In all samples in which photocatalysis was carried out using a mixture of Degussa P25 + PbS, Degussa P25 + WO_3_, and Degussa P25 + ZrO_2_, only one DP3 degradation product was detected. Additionally, no MET was detected at all after using the Degussa P25 + PbS mixture (this was the only sample in which complete MET degradation occurred). These results confirm the very good photocatalytic performance of this mixture for the removal of not only metronidazole but also most of its DPs. Figure 4 shows the proposed MET degradation pathways for heterogeneous photocatalysis.

Two DPs were detected with the same precursor ion [M + H]+ *m*/*z* = 172, identical as for the parent MET. According to the literature, MET in aqueous solutions tends to regroup the structure under radiation [42,43]. DP3 corresponds to the structure of 1-hydroxyethyl-2-methyl-4-hydroxyimino-5-oxo imidazole, which is formed in nitro to nitrite photolytic rearrangement reaction with the formation of a hydroxyimine group [68]. DP3 was detected in all samples except the dark sample, regardless of the catalyst used. DP4 is formed directly from DP3 when there is a complete rearrangement of the nitroimidazole ring into oxadiazole and loss of a water molecule [42]. DP4 was detected in 8 out of 15 samples analyzed, with the best Degussa P25 + PbS mixture detected only in the 60 min sample. The mass spectrum of DP2 corresponds to the regrouping product of metronidazole (DP4) following the cleavage of the methyl group from the oxadiazole ring, as confirmed by the data in the literature [44]. DP1 is commonly detected as a degradation product of MET under hydroxyl radical attack, and it is formed by the cleavage of the hydroxyethyl group from the nitroimidazole ring [69,70].

## 4. Conclusions

In this study, parameters were selected for the photocatalytic removal of MET in aqueous samples. The best catalyst proved to be a Degussa P25 + PbS mixture, for which the reaction rate constant was 0.0889 min^−1^, and the MET conversion was 95% in only 30 min. The Degussa P25 + WO_3_, ZnO + PbS, and Degussa P25 + ZrO_2_ mixtures also showed good photocatalytic performance, for which the MET removal efficiency in 60 min was as high as 94%. The highest photocatalysis efficiency was obtained at an irradiance of 1000 W m^−2^, where 95% of MET was removed in 30 min. The lowest MET removal efficiency (61.6%, 60 min) was obtained using an irradiance of 250 W m^−2^, corresponding to the average irradiance in Poland over a year. It was concluded that full conversion of MET using solar would require prolonging the photocatalysis time or using additional light sources.

The degree of sample mineralization determined by *TOC* changes was in the range of 36.0% (ZnO + WO_3_)–70.30% (Degussa P25 + PbS). Due to the discrepancy between the MET conversion of 95% and the MD of 70.3% in the model samples, four metronidazole degradation products were identified, and their structures were confirmed from data in the literature. The DPs were formed by rearrangement of the metronidazole ring and the C-C bond breaking in reaction with hydroxyl radicals.

The photocatalytic process procedure developed for the first time, using a Degussa P25 + PbS mixture with an irradiance of 1000 W m^−2^, allows efficient removal of METs from aqueous solutions. It should be emphasized that the effectiveness of the Degussa P25 + PbS mixture was tested in an aquatic environment, in a neutral reaction, in which PbS appears as an insoluble substance, whereby the potential environmental impact of PbS is substantially reduced. The use of a small amount of photocatalyst is attractive considering economic issues; however, the inability to use only sunlight as the irradiation source may significantly limit its application potential.

## Figures and Tables

**Table 1:** Constant rates of photocatalytic processes with the participation of various catalysts and their mixtures (catalyst concentration, 50 mg L^−1^; irradiance, 500 W m^−2^; irradiation time, 90 min).

Catalyst	k (1 min^−1^)	R^2^
**TiO_2_ + ZrO_2_**	0.0154	0.985
**TiO_2_**	0.0160	0.934
**TiO_2_ + PbS**	0.0164	0.976
**TiO_2_ + WO_3_**	0.0181	0.985
**WO_3_**	0.0215	0.997
**light**	0.0245	0.996
**ZnO**	0.0259	0.998
**PbS**	0.0275	0.997
**ZnO + WO_3_**	0.0278	0.997
**ZrO_2_**	0.0294	0.999
**ZnO + ZrO_2_**	0.0298	0.998
**Degussa P25**	0.0337	0.991
**P25 + ZrO_2_**	0.0337	0.994
**ZnO + PbS**	0.0338	0.987
**P25 + WO_3_**	0.0344	0.994
**P25 + PbS**	k_1_ = 0.0114 (0–20 min) k_2_ = 0.0889 (30–90 min)	0.991 (0–20 min) 0.993 (30–90 min)

**Table 2:** Influence of irradiation on the efficiency of degradation (catalyst, Degussa P25 + PbS (1:1; *w*/*w*); catalyst mass, 50 mg L^−1^; irradiation time, 90 min).

Time (min)	Removal Efficiency (%)			
	250 W m^−2^	500 W m^−2^	750 W m^−2^	1000 W m^−2^
0	0.00	0.00	0.00	0.00
5	6.75	10.2	11.6	14.3
15	15.6	22.1	30.4	52.1
20	17.6	28.3	43.3	71.5
30	28.4	53.6	69.5	95.0
35	30.6	65.0	82.7	100
40	35.1	77.5	91.3	100
45	41.3	87.7	94.1	100
60	61.6	96.1	100	100
75	75.2	100	100	100
90	86.3	100	100	100

**Table 3:** ***TOC*** value and the mineralization degree of samples after the photocatalysis process.

Sample	Time (min)	TOC (mg L^−1^)	SD (mg L^−1^)	Mineralization Degree (%)
**without light and catalyst**	90	28.20	0.23	0.0
**light**	90	18.25	0.02	37.1
**TiO_2_**	90	13.65	0.03	51.5
**ZnO**	90	11.63	0.04	58.7
**WO_3_**	90	11.03	0.29	60.8
**PbS**	90	10.53	0.03	62.6
**ZrO_2_**	90	10.79	0.03	61.7
**Degussa P25**	90	10.70	0.10	62.0
**P25 + PbS**	35	22.48	0.06	20.2
**P25 + PbS**	45	17.23	0.02	38.8
**P25 + PbS**	60	14.78	0.13	47.5
**P25 + PbS**	90	8.35	0.14	70.3
**P25 + ZrO_2_**	90	11.48	0.11	59.2
**P25 + WO_3_**	90	11.71	0.02	58.4
**ZnO + WO_3_**	90	18.02	1.04	36.0
**ZnO + ZrO_2_**	90	14.91	0.14	47.0
**ZnO + PbS**	90	15.31	0.06	45.6
**TiO_2_ + WO_3_**	90	13.17	0.03	53.2
**TiO_2_ + ZrO_2_**	90	14.06	0.03	50.1
**TiO_2_ + PbS**	90	12.51	0.01	55.6

## Data Availability

Data is contained within the article or supplementary material.

## Supplementary Materials

The following are available online, Figure S1: Spectrum of the xenon lamp using in the experiment depending on the light irradiance, Figure S2. Chromatogram of metronidazole standard substance obtained with LC-UV method, Figure S3. Effectiveness of metronidazole removal using TiO_2_ (anatase) and its mixtures, Figure S4. Effectiveness of metronidazole removal using Degussa P25 and its mixtures, Table S1. Effectiveness of metronidazole removal using ZnO and its mixtures, Table S2. Identified MET degradation products in mixtures after the photodegradation process.

## Sample Availability

Samples of the compounds are available from the authors.
