# Supplementary material for: Heterogeneous Photocatalysis of Metronidazole in Aquatic Samples
Source: Molecules. 2021 Dec 15;26(24):7612. doi: 10.3390/molecules26247612 (PMC8708392; doi:10.3390/molecules26247612)
Supplement: Supplementary file 1 [file molecules-26-07612-s001.zip › molecules-1489591-supplementary.pdf]

Heterogeneous photocatalysis of metronidazole in aquatic samples

Klaudia Stando[1,*], Patrycja Kasprzyk[1], Ewa Felis[2,3], Sylwia Bajkacz[1,2]

[1] *Silesian University of Technology, Faculty of Chemistry, Department of Inorganic Chemistry, Analytical Chemistry and Electrochemistry, B. Krzywoustego 6 Str., 44-100 Gliwice, Poland*

[2] *Silesian University of Technology, Centre for Biotechnology, ul. B. Krzywoustego 8, 44-100 Gliwice, Poland*

[3] *Silesian University of Technology, Faculty of Power and Environmental Engineering, Environmental Biotechnology Department, Akademicka 2 Str., 44-100 Gliwice, Poland*

SUPPLEMENTARY MATERIAL

In the supporting information was included:

Part 1. Xenon lamp spectra

Figure F1. Spectrum of the xenon lamp using in the experiment depending on the light irradiance

Part 2. Developing chromatographic conditions

Figure S1. Chromatogram of metronidazole standard substance obtained with LC-UV method

Selection of heterogenic photocatalysis conditions

Figure S2. Effectiveness of metronidazole removal using TiO_2 (anatase) and its mixtures

Table S1. Effectiveness of metronidazole removal using ZnO and its mixtures

Figure S3. Effectiveness of metronidazole removal using Degussa P25 and its mixtures

Identification of degradation products in post-reaction mixtures

Table S2. Identified MET degradation products in mixtures after the photodegradation process

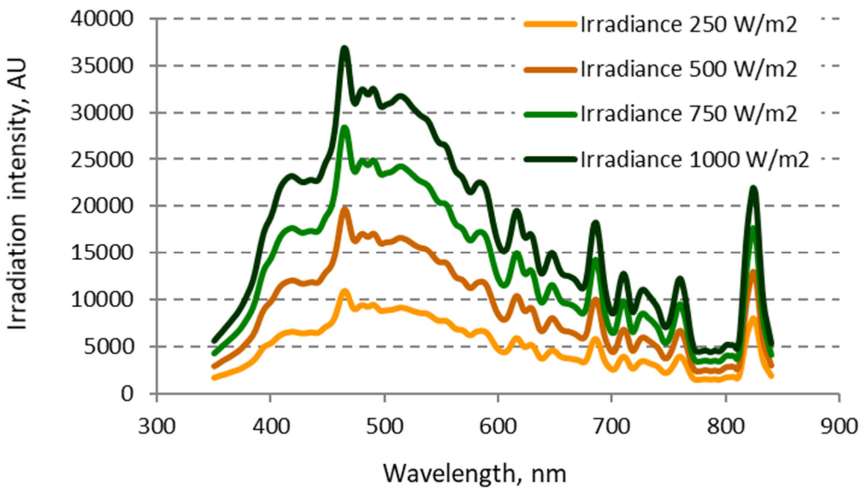

Figure S1. Spectrum of the xenon lamp using in the experiment depending on the light irradiance.

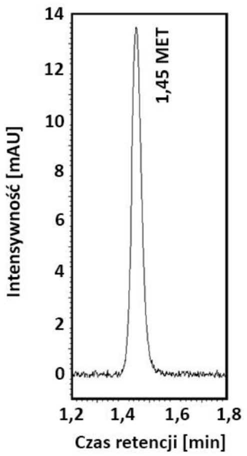

Figure S2. Chromatogram of metronidazole standard substance obtained with LC-UV method.

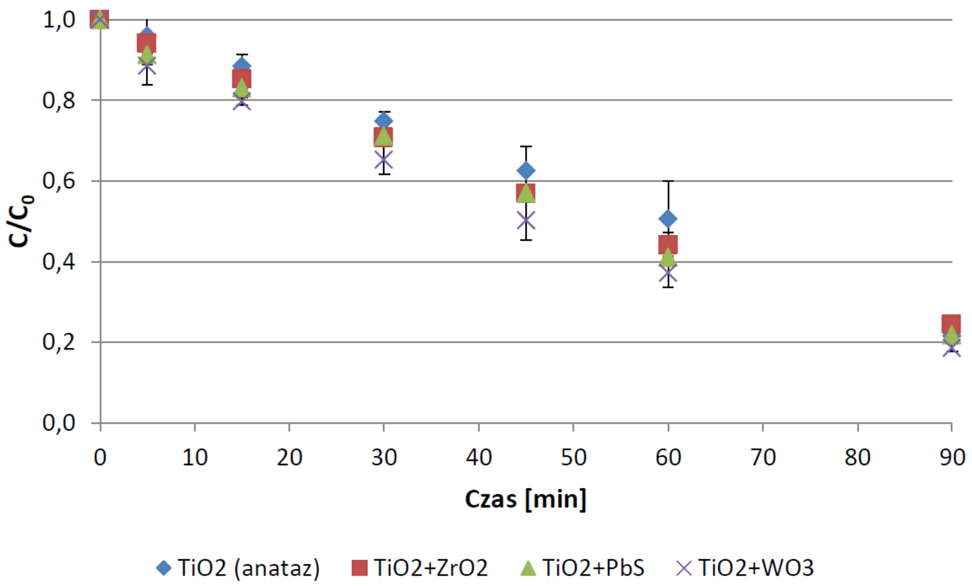

Figure S3. Effectiveness of metronidazole removal using TiO_2 (anatase) and its mixtures.

Table S1. Effectiveness of metronidazole removal using ZnO and its mixtures.

**Time (min)**	**Degradation rate (%)**			
	ZnO	$ZnO+ZrO_2$	ZnO+PbS	$ZnO+WO_3$
0	0.0	0.0	0.0	0.0
5	15.8	12.0	14.2	12.4
15	31.1	34.5	35.9	32.7
30	52.9	59.9	61.9	57.8
45	70.6	75.8	80.4	72.9
60	79.9	84.2	89.5	82.7
90	90.1	92.7	94.4	91.3

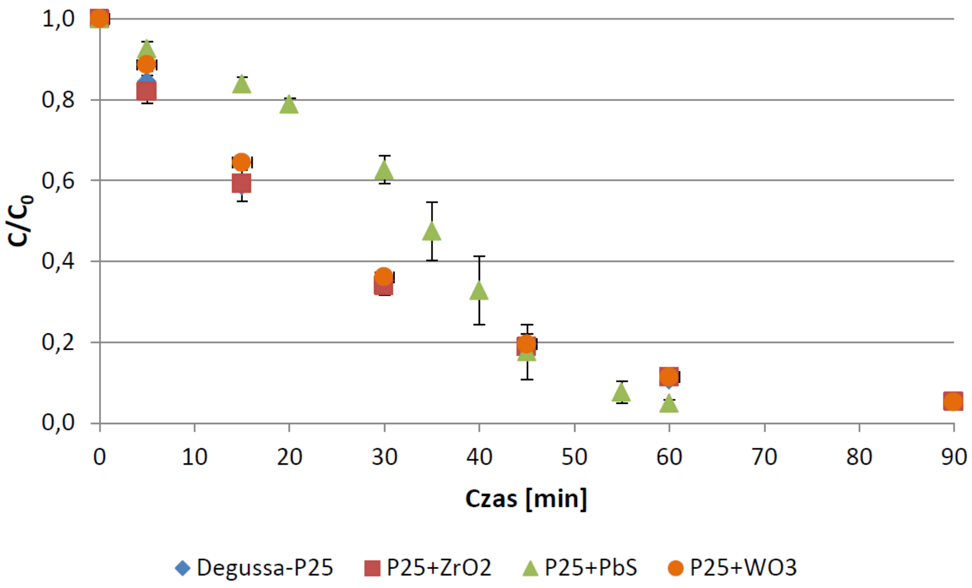

Figure S4. Effectiveness of metronidazole removal using Degussa P25 and its mixtures.

Table S2. Identified MET degradation products in mixtures after the photodegradation process.

**Sample**	**Time**	DP1	DP2	DP3	DP4	MET
		**Retention time (min)**				
		1.12	0.99	1.17	1.16	1.98
		**Precursor ion $[M+H]^+$ (m/z)**				
		128	158	172	172	172
TiO_2 (anataz)	90	+	-	+	+	+
MET+UVA	180	-	+	+	-	+
Degussa P25+PbS (bez światła)	60	-	-	-	-	+
Degussa P25+PbS	35	-	-	+	-	+
Degussa P25+PbS	45	-	-	+	-	+
Degussa P25+PbS	60	-	-	+	+	+
Degussa P25+PbS	90	-	-	+	-	+
Degussa P25+WO_3	90	-	-	+	-	+
Degussa P25+ZrO_2	90	-	-	+	-	-
TiO_2 (anataz)+WO_3	90	-	+	+	+	+
TiO_2 (anataz)+PbS	90	-	-	+	+	+
TiO_2 (anataz)+ZrO_2	90	-	-	+	+	+
ZnO+PbS	90	-	-	+	+	+
ZnO+ZrO_2	90	+	-	+	+	+
ZnO+WO_3	90	+	-	+	+	+
